# Implementation of a PMTCT programme in a high HIV prevalence setting in Johannesburg, South Africa: 2002–2015

**DOI:** 10.4102/sajhivmed.v21i1.1024

**Published:** 2020-03-23

**Authors:** Coceka N. Mnyani, Carol L. Tait, Remco P.H. Peters, Helen Struthers, Avy Violari, Glenda Gray, Eckhart J. Buchmann, Matthew F. Chersich, James A. McIntyre

**Affiliations:** 1 Department of Obstetrics and Gynaecology, School of Clinical Medicine, University of the Witwatersrand, Johannesburg, South Africa; 2 South African Centre of Epidemiological Modelling and Analysis (SACEMA), DST-NRF Centre for Excellence, Epidemiological Modelling and Analysis, Stellenbosch University, Stellenbosch, South Africa; 3 Anova Health Institute, Johannesburg, South Africa; 4 Department of Medical Microbiology, Maastricht University Medical Centre, Maastricht, The Netherlands; 5 Division of Infectious Diseases and HIV Medicine, Department of Medicine, University of Cape Town, Cape Town, South Africa; 6 Perinatal HIV Research Unit, Faculty of Health Sciences, University of the Witwatersrand, Johannesburg, South Africa; 7 Wits Reproductive Health and HIV Institute, Faculty of Health Sciences, University of the Witwatersrand, Johannesburg, South Africa; 8 School of Public Health and Family Medicine, University of Cape Town, Cape Town, South Africa

**Keywords:** PMTCT, South Africa, pregnant women living with HIV (PWLHIV), paediatric HIV infection, health systems

## Abstract

**Background:**

Great strides have been made in decreasing paediatric human immunodeficiency virus (HIV) infections, especially in sub-Saharan Africa. In South Africa, new paediatric HIV infections decreased by 84% between 2009 and 2015. This achievement is a result of a strong political will and the rapid evolution of the country’s prevention of mother-to-child transmission (PMTCT) guidelines.

**Objectives:**

In this paper we report on the implementation of a large PMTCT programme in Soweto, South Africa.

**Methods:**

We reviewed routinely collected PMTCT data from 13 healthcare facilities, for the period 2002–2015. Antiretroviral therapy (ART) coverage among pregnant women living with HIV (PWLHIV) and the mother-to-child transmission (MTCT) rate at early infant diagnosis were evaluated.

**Results:**

In total, 360 751 pregnant women attended the facilities during the review period, and the HIV prevalence remained high throughout at around 30%. The proportion of PWLHIV presenting with a known HIV status increased from 14.3% in 2009 when the indicator was first collected to 45% in 2015, *p* < 0.001. In 2006, less than 10% of the PWLHIV were initiated on ART, increasing to 88% by 2011. The MTCT rate decreased from 6.9% in 2007 to under 1% from 2013 to 2015, *p* < 0.001.

**Conclusion:**

The achievements in decreasing paediatric HIV infections have been hailed as one of the greatest public health achievements of our times. While there are inherent limitations with using routinely collected aggregate data, the Soweto data reflect progress made in the implementation of PMTCT programmes in South Africa. Progress with PMTCT has, however, not been accompanied by a decline in HIV prevalence among pregnant women.

## Background

Great strides have been made in the global fight to decrease new paediatric human immunodeficiency virus (HIV) infections. In 2011, the Joint United Nations Programme on HIV/AIDS (UNAIDS) launched the Global Plan to reduce new paediatric HIV infections by 90%, by 2015, with the baseline year being 2009.^1^ The target was to decrease the rate of mother-to-child transmission (MTCT) of HIV to 2% or less among non-breastfeeding women, and to 5% or less among breastfeeding women.^1^ As part of the Global Plan, 22 priority countries, which together accounted for 90% of the global number of pregnant women living with HIV (PWLHIV) in 2009, were identified for intensified efforts for the elimination of MTCT.^1^ India and 21 countries in sub-Saharan Africa made up the 22 priority countries.^1^

According to the 2016 UNAIDS report, there was a 60% decrease in the overall MTCT rate in 21 priority countries, from 22.4% in 2009 to 8.9% in 2015 (data on India were not available).^2^ The decrease in the MTCT rate is largely because of the increased coverage of more efficacious antiretroviral regimens for the prevention of mother-to-child transmission (PMTCT) of HIV.^2,3,4,5^ South Africa was identified as one of the 22 priority countries, and through strong political will and rapid evolution of the country’s PMTCT guidelines, new paediatric HIV infections decreased by 84% between 2009 and 2015, with an estimated 330 000 infections averted.^2^ The UNAIDS estimate for the MTCT rate for South Africa in 2015 was 2%. This was consistent with findings from a national survey conducted in 2012–2013, involving over 9000 infant–caregiver pairs, with an MTCT rate of 2.6% at 4–8 weeks.^2,6^

Donor funding has been critical in the establishment of the South African PMTCT and antiretroviral therapy (ART) programmes, with the country being the largest recipient of grants from the United States President’s Emergency Plan for AIDS Relief (PEPFAR).^7,8,9,10,11^ President’s Emergency Plan for AIDS Relief funding of HIV programmes in South Africa started in 2004, with direct service provision through the placement of staff and infrastructure in public healthcare facilities.^8,10^ From 2012, there was a transition in PEPFAR funding from direct service provision to technical support, with the South African government increasingly taking up ownership of the country’s HIV programme.^8,11^ By 2016, more than 75% of South Africa’s HIV response was funded by the government.^12^

This article reports on the outcomes of a large PMTCT programme in Soweto, South Africa, over time, including the coverage of ART among PWLHIV and the MTCT rate at approximately 6 weeks of age.

## Methods

### Study setting and design

We conducted a retrospective study of routinely collected PMTCT data from 13 public healthcare facilities that have been part of the Soweto PMTCT programme since its inception in 2002. Of the 13 facilities, one is a tertiary-level referral hospital (Chris Hani Baragwanath Academic Hospital), and 12 are primary healthcare facilities, of which six have delivery units. Soweto is an area of mixed urban and informal settlements, with an estimated population of approximately 1.7 million people.^13^

### History of the Soweto prevention of mother-to-child transmission programme

The Soweto PMTCT programme was established in 2000 as the Demonstration of Antiretroviral Treatment (DART) programme initiated by the Perinatal HIV Research Unit (PHRU).^14^ The programme was initially funded by the Elizabeth Glaser Paediatric AIDS Foundation (EGPAF) with funding from the United States Agency for International Development (USAID), the Fonds De Solidarité Thérapeutique International (FSTI) and the Gauteng Department of Health, and from 2004 it was funded by PEPFAR, through the USAID.^14^ As part of the DART programme, pregnant women were offered voluntary counselling and testing for HIV and, if found to be HIV-positive, were issued a single-dose nevirapine (NVP) to be taken intrapartum, and also a single-dose NVP to be given to the infant immediately after birth. A free 6-month supply of infant formula was also available for women living with HIV who elected not to breastfeed.

The programme evolved in line with changes in the South African PMTCT and ART guidelines, and since 2009, the programme has been supported by the Anova Health Institute (Anova), a USAID/PEPFAR-funded non-profit organisation. The donor-funded support was initially through direct service provision with placement of staff – doctors, professional nurses, data collectors and lay counsellors – in public health facilities working alongside government employees. There was also infrastructure, pharmacy, and monitoring and evaluation support for the facilities providing HIV services. With the PEPFAR funding transitioning to technical support, the focus in support shifted to mentoring and quality improvement of the programmes through monitoring and evaluation.

### Evolution of the South African prevention of mother-to-child transmission guidelines

Prior to 2002, no antiretroviral (ARV) prophylaxis or treatment was available in the South African public health sector, and ARVs were only available as part of research projects.^15,16,17^ From 2002 until 2007, only mother–infant single-dose NVP was available for PMTCT (Table 1). Additional zidovudine (AZT) monotherapy for PMTCT prophylaxis was introduced in 2008, initially started at 28 weeks’ gestation, and from 2010 at 14 weeks’ gestation.^18,19,20^ Antiretroviral therapy became available in South Africa in 2004 and the eligibility criterion was a CD4 count of < 200 cells/µL, or World Health Organization (WHO) stage 4 disease.^21^ CD4 count testing to assess ART eligibility became routinely available from 2005. The CD4 count threshold for ART initiation in pregnant women increased to ≤ 350 cells/µL in 2010.^20^

**TABLE 1:** Evolution of the South African prevention of mother-to-child transmission and antiretroviral therapy guidelines, 2002–2015.^18,19,20,21,23,24^

	2002	2004	2008	2010	2012	2013	2015
**PMTCT guidelines**	Intrapartum single-dose NVP	Intrapartum single-dose NVP	Option A – zidovudine monotherapy for PMTCT, for pregnant women not eligible for lifelong treatment	Integration of ART initiation within antenatal clinics	Withdrawal of free infant formula for WLHIV who elect not to breastfeed	Option B – triple therapy for all PWLHIV, with postpartum withdrawal of therapy in those not eligible for lifelong treatment	Option B+ – lifelong ART for all PWLHIV, regardless of level of immune suppression
**Infant prophylaxis**	Single-dose NVP after birth	Single-dose NVP after birth	7 days of AZT, or 28 days if the mother received less than 4 weeks of AZT or ART	6 weeks of NVP and extended for the duration of breastfeeding if mother not on ART	-	6 weeks of NVP and extended for the duration of breastfeeding if mother not on ART	6 weeks NVP and extended to 12 weeks if infant at high risk of MTCT†
**ART guidelines**	-	Triple therapy Eligibility criteria: CD4 < 200 cells/µL, or WHO stage 4 disease	Triple therapy Eligibility criteria: CD4 < 200 cells/μL, or WHO stage 4 disease	ART eligibility criteria for pregnant women: CD4 ≤ 350 cells/µL, or WHO stage 3 or 4 disease	ART eligibility criteria for pregnant women: CD4 ≤ 350 cells/μL, or WHO stage 3 or 4 disease	ART eligibility criteria for pregnant women: CD4 ≤ 350 cells/μL, or WHO stage 3 or 4 disease	ART for all PWLHIV, regardless of level of immune suppression

PMTCT, prevention of mother-to-child transmission of HIV; ART, antiretroviral therapy; AZT, zidovudine; WLHIV, women living with HIV; PWLHIV, pregnant women living with HIV; MTCT, mother-to-child transmission of HIV; NVP, nevirapine.

† , Infants at high risk of MTCT: mother had no ART or less than 4 weeks of ART during pregnancy and infant breastfed, HIV-positive diagnosis made during breastfeeding.

Up to September 2010, the antenatal clinics in the 12 primary healthcare facilities only provided antiretroviral prophylaxis for PMTCT, and PWLHIV who were eligible for lifelong ART were referred to a separate ART initiation site. In that time period, the only antenatal clinic that initiated ART was at Chris Hani Baragwanath Academic Hospital. At the primary health clinics, pregnant women diagnosed with HIV infection were referred to an ART initiation site, which could be in a different section of the same health facility, or in a different facility. Over a period of 18 months, beginning in October 2010, nurse-initiated and managed ART (NIMART) was introduced in the antenatal clinics, with PWLHIV receiving their antenatal and HIV care in the same facility. Nurse-initiated and managed ART, a task-shifting initiative to increase the number of patients initiated on ART, meant that professional nurses, including midwives, could initiate and manage patients on ART.^22^ Postpartum, the women were transitioned to adult HIV care for follow-up. From 2002 until 2011, a 6-month supply of free infant formula was available for all WLHIV who elected not to breastfeed.

In 2013, WHO Option B, where all pregnant and postpartum WLHIV were initiated on an efavirenz-based fixed-dose combination, was introduced.^23^ Treatment was stopped postpartum if not breastfeeding, or after cessation of breastfeeding, if the woman was not eligible for lifelong ART for her own health.^23^ The most recent PMTCT guideline change was in 2015 when all pregnant and postpartum women living with HIV became eligible for lifelong treatment regardless of CD4 count level, Option B+.^24^

The 2013 PMTCT guideline reinforced the recommendation that was first made in the 2010 guideline that pregnant women who initially tested HIV-negative were to be routinely retested during pregnancy at around 32 weeks’ gestation. For women who presented intrapartum with an unknown HIV status, or with a negative HIV test done prior to 32 weeks’ gestation, or done more than 12 weeks prior to delivery, the recommendation was for retesting intrapartum. Postpartum, the recommendation was to repeat the HIV test at 6 weeks postpartum and every 3 months during breastfeeding. In the 2015 guidelines, the recommendation is for routine repeat HIV testing every 3 months during pregnancy, intrapartum and postpartum as per the 2013 guidelines. Routine testing of HIV-exposed infants, using HIV polymerase chain reaction (PCR), became standard of care from 2004, and was offered at around 6 weeks of age. Also, as part of routine care, an HIV antibody test was done at 18 months in infants who initially tested negative at early infant diagnosis. Since 2015, routine testing of HIV-exposed infants was done at birth, 10 weeks of infant age, 6 weeks post-cessation of breastfeeding and at 18 months.

It is in this background of evolving guidelines and the changing focus of donor funding that we evaluated the Soweto PMTCT programme.

### Data management and analysis

As part of routine reporting to the District Health and Information System (DHIS), PMTCT data were collected on PWLHIV and HIV-exposed infants, and the indicators collected changed over time with evolving PMTCT guidelines. We extracted aggregate data from paper-based and electronic registers on core PMTCT indicators for the period 2002–2015. The indicators that we collected data for include the following: pregnant women presenting for their first antenatal visit and gestational age at first visit; pregnant women tested for HIV during pregnancy and those presenting already known to be living with HIV and whether already on ART; PWLHIV who were issued with single-dose NVP prophylaxis antenatally; PWLHIV who had a CD4 count done during pregnancy and those who had a CD4 count of < 200 cells/µL and ≤ 350 cells/µL; the number of ART-eligible pregnant women who were initiated on ART; and the number of HIV-exposed infants who were tested for HIV and those found to be HIV-positive.

The prevalence of human immunodeficiency virus among pregnant women was calculated using the number of pregnant women presenting at the first antenatal visit already known to be living with HIV and those newly diagnosed as HIV-positive during pregnancy as the numerator and the total number of first visits as the denominator. The proportion of ART-eligible pregnant women was calculated using the number of PWLHIV who had a CD4 count of < 200 cells/µL or ≤ 350 cells/µL, depending on the CD4 count threshold at the time, divided by the total number who had a CD4 count done. For the 2013–2015 data, all PWLHIV who were not on ART at the first antenatal visit were regarded as eligible for treatment, in line with the guidelines. The number of HIV-exposed infants who were found to be HIV-positive at around 6 weeks of age was used to calculate the MTCT rate, using as the denominator the number of HIV-exposed infants tested in the 13 facilities. Data were analysed using Stata® version 14.0 (Stata Corporation, College Station, TX, USA).

### Ethical consideration

The study was approved by the University of the Witwatersrand Human Research Ethics Committee (Reference No. M140461), and access to the facilities was granted by the Johannesburg Health District Office.

## Results

From January 2002 to December 2008, around 30 000 pregnant women presenting for their first antenatal visit were seen in the programme annually (Table 2). As services became decentralised, and more facilities started providing antenatal services, and PMTCT and ART care, the number of pregnant women seen in the 13 facilities decreased from 2009 onwards. Most pregnant women presented for antenatal care after 20 weeks’ gestation; the proportion doing so increased from 40.8% in 2010 when the indicator was first collected, to 51.3% in 2015, *p* < 0.001. There was also a progressive increase in pregnant women known to be living with HIV, presenting from 14.3% in 2009 to 45.0% 2015, *p* < 0.001. Human immunodeficiency virus testing rates during pregnancy were high throughout the study period, increasing from 95.8% in 2002 to 100% by 2010. The HIV prevalence was high from the beginning of the review period in 2002, with 28.9% of pregnant women found to be living with HIV, reaching a peak of 33.1% in 2009 (Table 2). From 2002 to 2007, when only single-dose NVP was available for PMTCT prophylaxis and issued at the first antenatal visit, the proportion of women who were issued prophylaxis peaked at 96.1% within 1 year of the implementation of the programme. Over time, the proportion of women who were issued NVP prophylaxis at the first antenatal visit decreased, as there was a shift to issuing the prophylaxis intrapartum.

**TABLE 2:** Antenatal human immunodeficiency virus testing and human immunodeficiency virus prevalence among pregnant women at 13 healthcare facilities in Soweto, 2002–2015.

Indicators†	2002		2003		2004		2005		2006		2007		2008		2009		2010		2011		2012		2013		2014		2015	
	*n*	%	*n*	%	*n*	%	*n*	%	*n*	%	*n*	%	*n*	%	*n*	%	*n*	%	*n*	%	*n*	%	*n*	%	*n*	%	*n*	%
First antenatal visits	30 064	-	28 840	-	30 066	-	30 291	-	30 739	-	30 090	-	30 174	-	28 652	-	23 663	-	21 709	-	20 490	-	20 324	-	17 951	-	17 698	-
First visits < 20 weeks	-	-	-		-		-		-	-	-	-	-	-	-	-	9643	40.8	8476	39.0	8196	40.0	8458	41.6	8365	46.6	9072	51.3
Known PWLHIV‡	-	-	-	-	-	-	-	-	-	-	-	-	-	-	1356	14.3	886	12.4	1120	18.1	1232	21.4	2255	35.4	2195	38.0	2192	45.0
Known PWLHIV on ART	-	-	-	-	-	-	-	-	-	-	-	-	-	-	-	-	591	66.7	907	81.0	1120	90.9	2056	91.2	1918	87.4	2064	94.2
Tested for HIV at first visit§	28 802	95.8	27 305	94.7	29 031	96.6	29 327	96.8	30 123	98.0	29 707	98.7	29 965	99.3	28 543	104.6	23 665	103.9	20 569	99.9	19 249	99.4	18 468	102.2	15 993	101.5	15 341	98.9
Diagnosed as PWLHIV	8316	-	7392	-	8917	-	8721	-	8872	-	8850	-	8774	-	8130	-	6243	-	5062	-	4536	-	4121	-	3575	-	2659	-
Total PWLHIV¶	8316	-	7392	-	8917	-	8721	-	8872	-	8850	-	8774	-	9486	-	7129	-	6182	-	5768	-	6376	-	5770	-	4851	-
HIV prevalence	28.9	-	27.1	-	30.7	-	29.7	-	29.5	-	29.8	-	29.3	-	33.0	-	30.1	-	28.5	-	28.2	-	31.4	-	32.1	-	27.4	-
Received single-dose nevirapine at antenatal visit ††	5966	71.7	7103	96.1	8295	93.0	7782	89.2	7704	86.8	7910	89.4	7966	90.8	6031	74.2	-	-	-	-	-	-	-	-	-	-	-	-

HIV, human immunodeficiency virus; PWLHIV, pregnant women living with HIV; ART, antiretroviral therapy.

† , Indicators changed as PMTCT guidelines evolved.

‡ , For known PWLHIV, the denominator is the total number identified as living with HIV.

§ , Represents aggregate data of the total number of pregnant women tested for HIV at the first antenatal visit, and with rates > 100%, indicates that there were pregnant women with known HIV status who had repeat testing.

¶ , Total PWLHIV = known and diagnosed as living with HIV at first visit; for HIV prevalence, the numerator is the total PWLHIV and the denominator the first antenatal visits.

†† , Some women received Option A.

- , Indicates that data for the indicator were not collected during that period.

**FIGURE 1:**
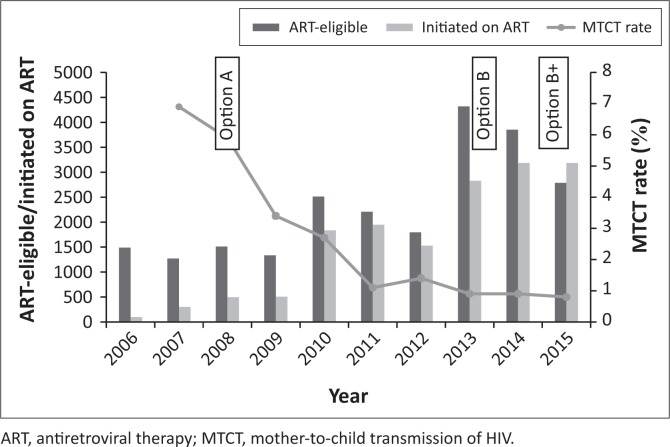
Eligible pregnant women initiated on ART and MTCT rate, 2006–2015.

With the CD4 count threshold for ART eligibility at < 200 cells/µL from 2005 to 2009, approximately 16% of PWLHIV were eligible for ART. When the CD4 count threshold was increased to ≤ 350 cells/µL in 2010, the proportion of ART-eligible pregnant women increased to around 40%. There was a progressive increase in the proportion of eligible women initiated on ART, from less than 10% in 2006 to over 80% by 2011 (Table 3 and Figure 1). From 2013, all PWLHIV were eligible to be started on ART regardless of the CD4 count level. Data for routine PCR testing of HIV-exposed infants, at around 6 weeks of age, are available for the period 2007–2015. A total of 41 948 PCR tests, with results, were reported, and of these, 1195 were found to be positive (Table 4). The MTCT rate at around 6 weeks of age decreased from 7.0% in 2007 to less than 1% in 2013–2015, *p* < 0.001 (Figure 1).

**TABLE 3:** Pregnant women living with human immunodeficiency virus assessed for antiretroviral therapy eligibility and initiated on treatment at 13 healthcare facilities in Soweto, 2005–2015.

Indicator	2005		2006		2007		2008		2009		2010		2011		2012		2013		2014		2015	
	*n*	%	*n*	%	*n*	%	*n*	%	*n*	%	*n*	%	*n*	%	*n*	%	*n*	%	*n*	%	*n*	%
Newly diagnosed HIV-positive	8721	†	8872	†	8850	†	8774	†	8130	†	6243	†	5062	†	4536	†	4121	†	3575	†	2659	†
Known PWLHIV not on ART	†	†	†	†	†	†	†	†	†	†	295	†	213	†	112	†	199	†	277	†	128	†
CD4 counts done†	4453	51.1	8767	98.8	8912	100.7	8775	100.0	8163	100.4	6741	103.1	5525	104.7	4811	103.5	4299	99.5	3694	95.9	†	†
CD4 < 200	719	16.2	1489	17.0	1272	14.3	1511	17.2	1353	16.6	973	14.4	906	16.4	740	15.4	†	†	†	†	†	†
CD4 ≤ 350	†	†	†	†	†	†	†	†	†	†	2513	37.3	2209	40.0	1795	37.3	†	†	†	†	†	†
Initiated on ART‡	†	†	96	6.5	300	23.6	495	32.8	505	37.3	1836	73.1	1949	88.2	1528	85.1	2830	65.5	3185	82.7	3187	114.3

HIV, human immunodeficiency virus; PWLHIV, pregnant women living with HIV; ART, antiretroviral therapy.

† , Represents aggregate data of the total number of CD4 counts’ first tests done.

‡ , Aggregate data on the total number initiated on ART and the denominator is the total number eligible for ART, based on the eligibility criteria at the time.

- , Indicates that data for the indicator were not collected during that period.

**TABLE 4:** Human immunodeficiency virus polymerase chain reaction testing of infants exposed to human immunodeficiency virus at around 6 weeks of age in the 13 healthcare facilities in Soweto, 2007–2015.

Indicator	2007	2008	2009	2010	2011	2012	2013	2014	2015
PCR done	4827	5529	5531	5380	4962	4648	3978	3765	3328
PCR positive	332	324	180	145	54	64	35	33	28
% PCR positive	6.9	5.9	3.3	2.7	1.1	1.4	0.9	0.9	0.8

HIV, human immunodeficiency virus; PCR, polymerase chain reaction.

## Discussion

In this 14-year review of the Soweto PMTCT programme, there was a progressive decline in the MTCT rate, at approximately 6 weeks of age, to under 1% by 2013. The decrease coincides with an increase in the proportion of PWLHIV initiated on ART, as the PMTCT guidelines evolved. Coverage of HIV testing of pregnant women was high throughout the study. The HIV prevalence remained high, with no discernible change over time. Donor funding and local non-governmental organisation (NGO) support were critical in the establishment of the Soweto PMTCT programme, and the collaboration with the South African Department of Health ensured sustainability of the programme.

While NVP and AZT monotherapy prophylaxis were important in decreasing the risk of MTCT in low-resource settings with limited access to ART, the greatest risk of transmission is in women with high viral loads who receive no or limited duration of ART during pregnancy.^25^ The decrease in the perinatal transmission rate in the Soweto programme became evident from 2008 to 2013, a period of rapid evolution of the South African PMTCT guidelines. The period saw the introduction of dual prophylaxis for PMTCT, an increase in the CD4 threshold for ART eligibility in pregnant women and also the introduction of NIMART within antenatal clinics.^19,20,23^ The decline in MTCT rate to below 2% coincided with the rapid increase in the number of ART-eligible pregnant women initiated on treatment in the programme. Integration of antenatal and HIV services, which was introduced in the programme in 2010, has been shown to increase the proportion of ART-eligible pregnant women initiated on ART.^26,27^ The infant HIV PCR testing reported does not reflect coverage, as testing was done in several other facilities in the Soweto area, not reported in this article. In spite of this, the trend in the decline in the MTCT rate is similar to that shown in published data in South Africa. It is also similar to the National Health Laboratory Services (NHLS) data for the Greater Soweto area which show a decline in the MTCT rate from 8.2% in 2007, to 1.6% in 2015 (personal communication, G. Sherman). While we did not have figures on breastfeeding transmission, postpartum MTCT remains a challenge with the 2016 UNAIDS report estimating that more than 50% of new HIV infections among children occur during the breastfeeding period.^2^

In spite of the gains made towards the elimination of MTCT in South Africa, several challenges remain.^28^ Only limited progress has been made in the prevention of new HIV infections among women of reproductive age, an important aspect of PMTCT.^2^ South Africa is reported to have had the highest number of new HIV infections among women of reproductive age globally in the period 2009–2015, and this is reflected in the high HIV prevalence among pregnant women, a consistent finding throughout the review period in our study.^2,29^ The finding of a consistently high HIV prevalence is similar to figures reported in the national antenatal sentinel HIV prevalence surveys that have been conducted in South Africa since 1990.^29^ Pregnant women in South Africa as a whole still present at an advanced gestational age for their first antenatal visit, with just over 50% reported to have presented before 20 weeks in 2014, albeit this being an increase from 36.7% in 2010.^5^ We found similar figures in our study.

The delayed presentation for antenatal care results in late ART initiation in those diagnosed as living with HIV during pregnancy. In our study, the majority of women were first diagnosed as living with HIV during their pregnancies, a finding reported in several studies conducted in South Africa and other sub-Saharan African countries, although this figure decreased over time.^30,31,32,33^ In spite of the late presentation for antenatal care, there was a steady increase in the proportion initiated on ART during pregnancy, a trend reported in published DHIS data.^34^ Among pregnant women who presented already known to be living with HIV, the proportion already on ART was high, a finding similar to that reported in the 2017 South African national antenatal sentinel HIV prevalence survey.^29^

One of the limitations of using aggregate data is that the denominators used to calculate rates for some of the indicators are proxy indicators. The data presented are the best available representation of the ideal, which would have been to have longitudinal data on each patient, from HIV diagnosis to the initiation of ART, and also have delivery details and linked infant HIV testing. The indicators collected also changed over time as guidelines changed. Details on the denominators used are presented in the ‘Results’ section. For HIV testing rates, no data were collected prior to 2009 on PWLHIV who already knew their HIV status and those who were already on ART. Hence, in this period, among those newly identified as HIV-positive, there will have been a proportion of PWLHIV who already knew their HIV status and may have been on treatment. Prior to 2013, for the indicators on PWLHIV assessed for ART eligibility and initiated on treatment, the numerator and denominator do not reflect the same group of women seen in 1 month, but the numbers even out over several months. Criteria for ART eligibility are reported in the ‘Results’ section. There were no data available on ART eligibility based on the WHO clinical staging.

There are also additional limitations with using routine, aggregate data, and these are related to the completeness and accuracy of the data.^35,36^ In their assessment of routinely collected PMTCT data from 57 public health facilities in South Africa, Nicol et al. raised concerns about the quality and consistency of reported data.^36^ The main discrepancies identified were between data in paper-based registers and the monthly facility reports.^36^ The discrepancies highlighted problems with data capturing related to lack of sufficient staff, and competence in recording and validation of data.^36^ In the Soweto PMTCT programme, there have always been dedicated data collectors and data managers involved in monitoring and evaluation of the programme. While recording of data in the facility registers remains primarily the responsibility of staff working at the healthcare facilities, data managers are involved in the validation of data and overseeing their work.

In spite of the limitations of the study, the Soweto PMTCT programme is a success story of the collaboration between donor-funded organisations and the South African Department of Health. The strength of this study is that it reports on a large PMTCT programme, over a long review period. While there are inherent inaccuracies with routinely collected aggregate data, the trends reported in this article are similar to those reported in published DHIS data and national surveys in South Africa.^29,34,37^ To our knowledge, no PMTCT data of this magnitude have been published from a low-resource, high HIV prevalence setting. Data from the programme illustrate that it is possible to significantly decrease the MTCT rate even in a high HIV prevalence setting. It is important to ensure that the gains made towards elimination of MTCT are sustained beyond the immediate postpartum period, and also ensure that HIV-exposed uninfected infants survive and thrive.^38^ There also needs to be a concerted effort to decrease the rate of new HIV infections, especially among women of reproductive age.

## References

[1] Joint United Nations Programme on HIV/AIDS (UNAIDS) Countdown to zero: Global plan towards the elimination of new HIV infections among children by 2015 and keeping their mothers alive, 2011–2015. Geneva: UNAIDS; 2011.

[2] Joint United Nations Programme on HIV/AIDS (UNAIDS) On the fast-track to an AIDS-free generation The incredible journey of the global plan towards the elimination of new HIV infections among children by 2015 and keeping their mothers alive. Geneva: UNAIDS; 2016.

[3] Burton R, Giddy J, Stinson K Prevention of mother-to-child transmission in South Africa: An ever-changing landscape. Obstet Med. 2015;8(1):5–12. 10.1177/1753495X15570994 27512452 PMC4934997

[4] Bhardwaj S, Barron P, Pillay Y, et al Elimination of mother-to-child transmission of HIV in South Africa: Rapid scale-up using quality improvement. S Afr Med J. 2014;104(3):239–243. 10.7196/SAMJ.7605 24893500

[5] Millennium Development Goals 5: Improve maternal health [homepage on the Internet]. Pretoria: Statistics South Africa; 2015 [cited 2017 Jun]. Available from: http://www.statssa.gov.za/MDG/MDG_Goal5_report_2015_.pdf

[6] Goga AE, Dinh TH, Jackson DJ, et al South Africa PMTCT Evaluation (SAPMCTE) Team. Population-level effectiveness of PMTCT option A on early mother-to-child (MTCT) transmission of HIV in South Africa: Implications for eliminating MTCT. J Glob Health. 2016;6(2):020405 10.7189/jogh.06.020405 27698999 PMC5032343

[7] Johnson K The politics of AIDS policy development and implementation in post-apartheid South Africa. AfricaToday. 2004;51(2):107–128. 10.1353/at.2005.0007

[8] Katz IT, Bassett IV, Wright AA PEPFAR in transition – Implications for HIV Care in South Africa. N Engl J Med. 2013;369(15):1385–1387. 10.1056/NEJMp1310982 24106930 PMC3897549

[9] Simelela NP, Venter WD A brief history of South Africa’s response to AIDS. S Afr Med J. 2014;104(3):249–251. 10.7196/SAMJ.7700 24893502

[10] Kavanagh MM The politics and epidemiology of transition: PEPFAR and AIDS in South Africa. J Acquir Immune Defic Syndr. 2014;65(3):247–250. 10.1097/QAI.0000000000000093 24346642

[11] Strauss M, Surgey G, Cohen S, for the South African National AIDS Council A review of the South African Comprehensive HIV and AIDS Grant [homepage on the Internet]. 2015 [cited 2017 Jun]. Available from: http://sanac.org.za/wp-content/uploads/2015/11/A-review-of-the-South-African-Conditional-Grant-for-HIV-30Mar-vMS.pdf

[12] PEPFAR investing more than $410 Million towards an AIDS-Free generation in South Africa [homepage on the Internet]. Media Advisory. 2016 [cited 2017 Jun]. Available from: https://za.usembassy.gov/pepfar-investing-410-million-towards-aids-free-generation-south-africa/

[13] Population of cities in South Africa [homepage on the Internet]. 2017 [cited 2018 Oct]. Available from: http://worldpopulationreview.com/countries/south-africa-population/cities/

[14] At the cutting edge of HIV/AIDS research: A review of the Perinatal HIV Research Unit, University of the Witwatersrand, 1996–2005 [homepage on the Internet]. [cited 2018 Oct]. Available from: https://www.phru.co.za/images/documents/phru_overview.pdf

[15] Gray GE, Urban M, Chersich MF, et al A randomized trial of two postexposure prophylaxis regimens to reduce mother-to-child HIV-1 transmission in infants of untreated mothers. AIDS. 2005;19(12):1289–1297. 10.1097/01.aids.0000180100.42770.a7 16052084

[16] Moodley D, Moodley J, Coovadia H, et al A multicentre randomized controlled trial of nevirapine versus a combination of zidovudine and lamivudine to reduce intrapartum and early postpartum mother-to-child transmission of human immunodeficiency virus type 1. J Infect Dis. 2003;187(5):725–735. 10.1086/367898 12599045

[17] Petra Study Team Efficacy of three short-course regimens of zidovudine and lamivudine in preventing early and late transmission of HIV-1 from mother to child in Tanzania, South Africa, and Uganda (Petra study): A randomised, double-blind, placebo-controlled trial. Lancet. 2002;359(9313):1178–1186. 10.1016/S0140-6736(02)08214-4 11955535

[18] Doherty T, Besser M, Donohue S, et al An evaluation of the prevention of mother-to-child transmission (PMTCT) of HIV initiative in South Africa – Lessons and key recommendations [homepage on the Internet]. Durban: Health Systems Trust: 2003 [cited 2018 Oct]. Available from: http://www.hst.org.za/sites/default/files/pmtct_national.pdf

[19] National Department of Health Policy and guidelines for the implementation of the PMTCT Programme. Pretoria: South African National Department of Health; 2008.

[20] National Department of Health, South Africa; South African National AIDS Council Clinical guidelines: PMTCT (prevention of mother-to-child transmission), 2010 [homepage on the Internet]. Pretoria: South African National Department of Health; 2010 [cited 2018 Oct]. Available from: http://www.sahivsoc.org/upload/documents/NDOH_PMTCT.pdf

[21] National antiretroviral treatment guidelines [homepage on the Internet]. Pretoria: Department of Health; 2004 [cited 2018 Oct]. Available from: http://www.doh.gov.za/docs/facts/2004/intro.pdf

[22] Colvin CJ, Fairall L, Lewin S, et al Expanding access to ART in South Africa: The role of nurse-initiated treatment. S Afr Med J. 2010;100(4):210–212. 10.7196/SAMJ.4124 20459957

[23] Revised antiretroviral treatment guideline update for frontline clinical health professionals [homepage on the Internet]. Pretoria: South African National Department of Health; 2013 [cited 2018 Oct]. Available from: http://www.sahivsoc.org/upload/documents/FDC%20Training%20Manual%2014%20March%202013(1).pdf

[24] National Consolidated Guidelines for the prevention of mother-to-child transmission of HIV (PMTCT) and the management of HIV in children, adolescents and adults [homepage on the Internet]. Pretoria: South African National Department of Health; 2015 [cited 2018 Oct]. Available from: http://www.sahivsoc.org/upload/documents/HIV%20guidelines%20_Jan%202015.pdf

[25] Townsend CL, Byrne L, Cortina-Borja M, et al Earlier initiation of ART and further decline in mother-to-child HIV transmission rates, 2000–2011. AIDS. 2014;28:1049–1057. 10.1097/QAD.0000000000000212 24566097

[26] Killam WP, Bushimbwa TC, Namwinga C, et al Antiretroviral therapy in antenatal care to increase treatment initiation in HIV-infected pregnant women: A stepped-wedge evaluation. AIDS. 2010;24(1):85–91. 10.1097/QAD.0b013e32833298be 19809271

[27] Stinson K, Jennings K, Myer L Integration of antiretroviral therapy services into antenatal care increases treatment initiation during pregnancy: A cohort study. PLoS One. 2013;8(5):e63328 10.1371/journal.pone.0063328 23696814 PMC3656005

[28] Luzuriaga K, Mofenson LM Challenges in the elimination of paediatric HIV-1 infection. N Engl J Med. 2016;374:761–770. 10.1056/NEJMra1505256 26933850

[29] Woldesenbet SA, Kufa T, Lombard C, et al The 2017 National Antenatal Sentinel HIV Survey, South Africa. National Department of Health [homepage on the Internet]. 2019 [cited 2019 Jul]. Available from: http://www.nicd.ac.za/wp-content/uploads/2019/07/Antenatal_survey-report_24July19.pdf

[30] Wettstein C, Mugglin C, Egger M, et al Missed opportunities to prevent mother-to-child-transmission: Systematic review and meta-analysis. AIDS. 2012;26(18):2361–2373. 10.1097/QAD.0b013e328359ab0c 22948267 PMC3741537

[31] Technau KG, Kalk E, Coovadia A, et al Timing of maternal HIV testing and uptake of prevention of mother-to-child transmission interventions among women and their infected infants in Johannesburg, South Africa. J Acquir Immune Defic Syndr. 2014;65(5):e170–e178. 10.1097/QAI.0000000000000068 24759066 PMC3999509

[32] Kim MH, Ahmed S, Hosseinipour MC, et al The impact of Option B+ on the antenatal PMTCT cascade in Lilongwe, Malawi. J Acquir Immune Defic Syndr. 2015;68:e77–e83. 10.1097/QAI.0000000000000517 25585302 PMC4359035

[33] Herce ME, Mtande T, Chimbwandira F, et al Supporting option B+ scale up and strengthening the prevention of mother-to-child transmission cascade in central Malawi: Results from a serial cross-sectional study. BMC Infect Dis. 2015;15:328 10.1186/s12879-015-1065-y 26265222 PMC4533797

[34] Massyn N, Peer N, English R, Padarath A, Barron P, Day C, editors District health barometer 2015/16. Durban: Health Systems Trust; 2016.

[35] Barron P, Pillay Y, Doherty T, et al Eliminating mother-to-child HIV transmission in South Africa. Bull World Health Organ. 2013;91(1):70–74. 10.2471/BLT.12.106807 23397353 PMC3537246

[36] Nicol E, Dudley L, Bradshaw D Assessing the quality of routine data for the prevention of mother-to-child transmission of HIV: An analytical observational study in two health districts with high HIV prevalence in South Africa. Int J Med Inform. 2016;95:60–70. 10.1016/j.ijmedinf.2016.09.006 27697233

[37] Sherman GG, Mazanderani AH, Barron P, et al Toward elimination of mother-to-child transmission of HIV in South Africa: How best to monitor early infant infections within the Prevention of Mother-to-Child Transmission Program. J Glob Health. 2017;7(1):010701 10.7189/jogh.07.010701 28567281 PMC5441442

[38] Evans C, Jones CE, Prendergast AJ HIV-exposed, uninfected infants: New global challenges in the era of paediatric HIV elimination. Lancet Infect Dis. 2016;16(6):e92–e107. 10.1016/S1473-3099(16)00055-4 27049574

